# Knowledge, attitude and premarital screening practices for sickle cell disease among young unmarried adults in an urban community in Lagos, Nigeria

**DOI:** 10.11604/pamj.2022.42.8.27705

**Published:** 2022-05-06

**Authors:** Esther Oluwakemi Oluwole, Chibuike Davidson Okoye, Adedoyin Oyeyimika Ogunyemi, Olusola Festus Olowoselu, Olufemi Abiola Oyedeji

**Affiliations:** 1 Department of Community Health and Primary Care, College of Medicine, University of Lagos, Lagos, Nigeria,; 2 Department of Haematology and Blood Transfusion, College of Medicine, University of Lagos, Lagos, Nigeria

**Keywords:** Sickle cell disease, knowledge, attitude, premarital screening practices, young unmarried adults, communities, Nigeria

## Abstract

**Introduction:**

sickle cell disease (SCD) refers to a group of inherited blood disorders that are life-long and affect many people globally. An estimate of 2.3% of the Nigerian population suffer from SCD and about 25% of adults have the sickle cell gene. Premarital screening for sickle cell gene is considered one of the methods of preventing new births of children with SCD among the young adults. The study assessed the knowledge, attitude, willingness to take premarital screening test for SCD and factors influencing knowledge among young unmarried adults in an urban community in Lagos, Nigeria.

**Methods:**

the study was cross-sectional descriptive among 300 respondents who were selected using multistage sampling technique. Data were collected using a pre-tested, interviewer-administered questionnaire and analyzed using SPSS software, version 25. Univariate and bivariate analysis were conducted with level of significance at p ≤ 0.05.

**Results:**

the mean age of respondents was 21.2± 3.5 years, and most 188 (62.7%) were males. About 139 (46.3%) and 165 (55.0%) of the respondents respectively had good knowledge and positive attitude towards SCD and premarital screening. Only 43% of the respondents knew their haemoglobin phenotype, however, majority (92.4%) were willing to have Hb phenotype test done. Knowledge of SCD and premarital screening was statistically significant with age, level of education and occupation of respondents (p<0.001).

**Conclusion:**

this study found less than half of the respondents with good knowledge, about half had positive attitude and poor premarital screening practices of SCD. Therefore, community-based health education and awareness programs on SCD and premarital screening among young adults is recommended.

## Introduction

Sickle cell disease (SCD) refers to a group of inherited blood disorders that are life-long and affect many people globally. It is the first “molecular disease,” caused by a single gene mutation [1]. It is an autosomal recessive genetic disorder where an individual who is heterozygous or carrier is referred to as having sickle cell trait (SCT), while those with homozygous or compound heterozygous for the mutation have SCD. People with SCD have an abnormal type of hemoglobin, which polymerizes when deoxygenated, causing the red blood cells to become sickle and rigid [2,3]. SCD affects all races and is commoner among people of African descent from tropical and subtropical regions where malaria is or was common. Also affected are people from Mediterranean Sea, Middle East and South India [2-4]. The two most frequent types of SCD are Sickle cell anemia (SCA) and Hemoglobin SC disease (HbSC) and both are hereditary diseases with an autosomal recessive pattern of inheritance. SCA occurs as a result of a single point mutation on the β-globin gene called S or Sickle hemoglobin (HbS), while HbSC is caused by the presence of two variants, resulting from HbS and HbC allelic genes in beta globin locus in chromosome 11p 15.5 [5]. People with SCD experience lifelong complications including anemia, infections, stroke, tissue damage, organ failure, intense painful episodes, and even premature death as a result of the mutation [1]. These debilitating consequences of SCD and the continuous treatment often limit the education, career opportunities, and quality of life of people living with SCD [1].

Nigeria has the highest prevalence of SCD worldwide. About 2.3% of the population suffer from SCD and about 25% of adults have the sickle cell gene, while the Hb C trait is found in about six percent of the Yoruba people of south-western Nigeria [4,6-8]. Over 300,000 babies are born worldwide with SCD annually, mostly in low- and middle-income countries with the majority of these births in Africa, about 150,000 of these births occur in Nigeria with a birth incidence of SCD of 20 per 1000 live births and about 100,000 of these children die [9,10]. SCD constitutes a major public health problem among the black race [7]. A study on knowledge and attitude to sickle cell disease among new graduates of Nigerian tertiary educational institutions reported severely deficient knowledge on the transmission of SCD among the graduates [11]. Another study conducted among youth in Lagos, Nigeria found that 13.4% of the respondents showed a negative attitude towards SCD and premarital Counselling [8]. Another study in Nigeria found that 69% of respondents had poor knowledge of SCD, while 95% had favorable attitude towards premarital screening [7]. SCD is a lifetime disease and a cause of severe morbidity that often require prolonged hospital admission with high mortality rate. With the relatively high prevalence of SCD in Nigeria and the potential to increase, prevention of SCD is of utmost importance among unmarried adults in urban areas, especially with the rise in premarital sex and delay in marriage [8]. In Nigeria, premarital genetic Counselling is voluntary, however, premarital screening for the sickle cell gene is considered one of the methods of preventing new births of children with SCD [8]. SCD is preventable, especially among the young unmarried adults who may start procreation soonest. But due to lack of knowledge of SCD, and unwillingness to undergo premarital screening for SCD, the prevalence of SCD might be on the increase. The study, therefore, aimed to assess the knowledge, attitude and willingness to undergo premarital screening for SCD among young unmarried adults in an urban community in Lagos, Nigeria. The findings of this study may provide evidence-based information which could be used by policymakers and stakeholders in designing targeted programs for young adults in communities.

## Methods

**Study location:** Alimosho is one of the twenty Local Government Area (LGA) in Lagos State, Nigeria. It is the largest local government in Lagos, with 1,319,571 inhabitants according to the official 2006 Census. It has six subdivisions as local council developments areas (LCDAs); Agbado/Oke-odo, Ayobo/Ipaja, Egbe/Idimu, Ikotun/Igando, Mosan Okunola and Alimosho LGA [12].

### Study design and sample size determination

This was a descriptive cross-sectional study conducted among 300 young unmarried persons in Alimosho LGA of Lagos. Sample size was calculated using a Cochran formula


$$n=\frac{z^{2}pq}{d^{2}}$$

Where n was the estimated minimum sample size; z-level of significance at 95% confidence level (1.96); p-proportion of respondents (0.80) with good knowledge of SCD from a similar study carried out in Lagos state, Nigeria [8] q=(1-p), d=level of precision (0.05%). The calculated minimum sample size was 246, which was increased by 20% to 300 to allow for incomplete questionnaires. Respondents who had spent at least six months in the community and never married were selected. Those who were ill, or failed to give informed consent, were excluded.

### Sampling technique

A multi-stage probability sampling technique was used to select the respondents from the study population in six stages. Stage 1 comprised of selection of two LCDAs (Ikotun/Igando and Mosan Okunola) by simple random sampling using the ballot method. In stage two, three wards were selected from each of the selected LCDA by simple random sampling via ballot. From Igando/Ikotun LCDA; Akesan, Egan and Ijegun wards were selected while from Mosun Okunola LCDA; Abesan, Okunola and Gowon estate wards were selected. Stage three comprised the selection of five streets from each of the selected wards by simple random sampling via ballot, while in stage four, ten houses were selected from each of the streets. The first house was selected by simple random sampling through ballot method, while subsequent houses were selected, using a systematic sampling method with sampling interval (k) derived from the formula; (k)=number of houses/number of houses required. Stage five comprised of selection of one household from each of the selected houses by simple random sampling via ballot. When the selected household had no eligible respondent, the next household was selected. In stage six, one respondent was selected from each household by simple random sampling via ballot. Only young unmarried adults who voluntarily gave their consent participated in the study. Data collection was from July 2018 to January 2019.

**Data collection tool:** a pretested interviewer-administered questionnaire was used to obtain data from respondents. The questionnaire was adapted from previous studies and modified accordingly [8,13-15]. The questionnaire was written in English language and divided into four sections. Section A consisted of socio-demographic characteristics of the respondents, Section B consisted of 20 questions on knowledge of the respondents about sickle cell disease and premarital screening while section C had 10 questions which assessed attitude of the respondents towards SCD and premarital screening on a five-point Likert´s scale as agreed, strongly agreed, neutral, disagreed and strongly disagreed. The fourth section assessed questions that determined the preventive practices of the respondents against SCD. The tool was pretested among thirty young unmarried persons in another local government area, which has a similar setting to the study area for accuracy and adequacy.

### Data analysis

Data were analyzed with Statistical Package for Social Sciences (SPSS) version 25 software. For knowledge questions, correct answers were scored one point, incorrect and don´t know scored zero point. The minimum score was 0 and the highest score was 20, while for attitude, the minimum score was 10 and the maximum was 50. The mean and median knowledge scores were assessed. Based on the mean score as the cut-off point, level of knowledge was classified into poor and good. Also, the attitude scores were classified into positive and negative attitude based on the median score. Categorical variables were presented as percentages or proportions, while continuous variables were presented as mean ± standard deviation (SD). Chi square was used to determine the association between categorical variables, and level of significance was set at p ≤ 0.05. Ethical approval for the study was obtained from the Health Research and Ethics Committee of Lagos University Teaching Hospital (ADM/DCST/HREC/APP/408) Written informed consent was obtained from each respondent with assurance of confidentiality of information and their right to withdraw from the study at any point in time. They were made to understand that involvement was voluntary.

## Results

**Socio-demographic characteristics of respondents:** a total of 300 adults who gave consent to participate in the study were interviewed. The respondents´ ages ranged from 15 to 34 years, with a mean of 21.2 ± 3.5 years. Most (63%) were males, about 68% were students. A higher percentage of the respondents (77%) had secondary level of education completed and 79% were Christians (Table 1).

**Table 1:** socio-demographic characteristics of study respondents, recruited from an urban community in Lagos, Nigeria from July 2018 to January 2019 (N=300)

Socio-demographic	Frequency (n=300)	Percentage (%)
**Age group (years)**		
<19	109	36.3
20-30	188	62.7
≥ 31	3	1.0
Mean age	21.2 ± 3.5 years	
**Gender**		
Female	112	37.3
Male	188	62.7
**Level of education**		
Primary	10	3.3
Secondary	230	76.7
Tertiary	60	20.0
**Occupational status**		
Students	205	68.3
Business and traders	51	17.1
Professionals	29	9.7
Unemployed/others	15	4.9
**Religion**		
Christianity	236	78.7
Islam	64	21.3

**Respondent´s knowledge and premarital screening of Sickle Cell Disease:** majority (85%) of the respondents knew that SCD is a blood disorder. Most (60% and 67%) knew SCD is hereditary and affects all age groups, respectively. However, close to half (48.3%) felt sickle cell trait (SCT) could change into sickle cell disease (SCD) overtime, and 45%, 36%, and 41% felt SCD can be transmitted by direct body contact, mosquito bite and act of witches/wizards respectively. About 34% and 21% thought that father or mother only could transmit the SCD gene to the child, respectively. Most (61%) of the respondents thought that SCD can be cured with medication. Over three-quarter of respondents (77.3%) knew that premarital screening could be done to rule out SCD or SCT before marriage, while 75% knew that screening should be done before marriage and 72% knew that the screening prevents having a child with SCD. Overall, less than half 139 (46.3%) of the respondents had good knowledge of SCD and premarital screening, with a mean knowledge score of 13.4± 3.2 out of 20 questions (Table 2).

**Table 2:** knowledge of sickle cell disease among young unmarried adults in an urban community in Lagos, Nigeria, recruited between July 2018 to January 2019 (N=300)

Knowledge Statements (ONLY CORRECT answers)	Frequency (n=300)	Percentage (%)
SCD is a blood disorder (True)	256	85.3
SCD is a hereditary disorder (True)	182	60.0
SCD affects all age groups (True)	200	66.7
SCT can change into SCD overtime (False)	114	38.0
SCD can be transmitted by direct body contact (False)	149	49.7
SCD can be transmitted by mosquito bite (False)	181	60.3
SCD an be transmitted caused by the act of witches/wizards (False)	160	53.3
Father only can transmit the SCD gene to the child (False)	160	53.3
Mother only transmits the SCD gene to the child (False)	199	66.0
If one parent is SS/SC and the other AA, all children will either be AS/AC (True)	224	74.7
If both parents have either AS/AC, each child has 25% chance of being SS/SC (True)	188	62.7
If one parent has SS/SC and the other AS/AC, baby has a 50% chance of being either SS/SC or AS/AC (True)	203	67.7
Severe body pain and yellowness of the eyes are common symptoms of SCD(True)	229	76.3
SCD can be cured with medication (False)	91	30.0
Premarital screening (PS) is done to rule out SCD or SCT before marriage (True)	232	77.3
PS should be done before marriage (True)	227	75.0
PS should be done after marriage (False)	204	68.0
PS should be done immediately after child delivery (False)	187	62.3
PS prevents having a child with SCD (True)	218	72.0
PS has no benefit (False)	251	83.0

**Respondents' attitude towards sickle cell disease prevention and premarital sickle cell screening:** less than half of the respondents (38.3%) agreed that relationship should be ended if discovered that genotypes predispose two people to having children with SCD, while a quarter (25%) were neutral. However, more than half (58.3%) agreed that one can be friends with a person living with SCD. Most (74.3%) of respondents felt people with SCD should not be isolated, about 67% seemed it good for everybody to know their genotype before marriage and 72% said it is important for two people in relationship to undergo genetic counselling before marriage. Overall, only 165 (55.0%) of the respondents had a positive attitude towards people with SCD and premarital screening, with a median score (IQR) of 35.0 (31.0-39.0) out of 50 points (Table 3).

**Table 3:** attitude towards sickle cell disease prevention and premarital sickle cell screening among young unmarried adults in an urban community in Lagos, Nigeria, recruited between July 2018 to January 2019 (N=300)

Attitude statements	SA (%)	A (%)	N (%)	D (%)	SD (%)
Relationship should be ended if discover that genotypes predispose two people to having children with SCD	49 (16.3)	66 (22.0)	75 (25.0)	65 (21.7)	45 (15.0)
Someone can be friends with a person living with SCD	78 (26.0)	97 (32.3)	33 (11.0)	49 (16.3)	43 (14.3)
People living with SCD can be invited to birthday parties	67 (22.3)	107(35.7)	31 (10.3)	57 (19.0)	38 (12.7)
One can eat with persons living with SCD	68 (22.7)	85 (28.3)	48(16.0)	67 (22.3)	32 (10.7)
One can work with people living with SCD	66 (22.0)	119 (39.7)	43 (14.3)	51 (17.0)	21 (7.0)
People with SCD should not be isolated	124 (41.3)	99 (33.0)	25(8.3)	34 (11.3)	18 (6.0)
It is good for everybody to know their genotype before marriage	113 (37.7)	88 (29.3)	10(3.3)	36 (12.0)	53 (17.7)
It is important for two people in relationship to undergo genetic counselling before marriage	103 (34.3)	113 (37.7)	22(7.3)	30 (10.0)	32 (10.7)
Premarital sickle cell test is necessary before agreement to marry	101 (33.7)	119 (39.7)	17(5.7)	37 (12.3)	26 (8.7)
I will go for premarital sickle cell screening before marriage	131 (43.7)	97 (32.3)	8(2.7)	37(12.3)	27 (9.0)

* SA= Strongly agree, A= Agree, N= Neutral, D= Disagree, SD= Strongly disagree

**Pre-marital screening practices of respondents:** less than half (43%) of the respondents knew their haemoglobin phenotype, most (46%) took the test because of school entry and majority (80%) were of Hb AA. About 35% of the respondents who did not know their haemoglobin phenotype felt they were not getting married soon, while 23% said they didn´t know about the test at all. However, a majority (92.4%) showed willingness to have their Hb phenotype test carried out before going into marriage (Table 4).

**Table 4:** pre-marital screening practices of young unmarried adults in an urban community in Lagos, Nigeria, recruited between July 2018 to January 2019 (N=300)

Variables	Frequency (n=300)	Percentage (%)
**Aware of self-genotype**		
Yes	129	42.9
No	171	57.1
**Reason for genotype test (n=129)**		
School entry	59	45.7
Infancy	33	25.6
Curiosity	26	20.2
Doctors request	11	8.5
**Genotype stated (n=129)**		
AA	103	79.8
AS	26	20.2
**Reasons for not knowing genotype (n=171)**		
Not getting married soon	60	35.1
Don´t know about it	40	23.4
No money	24	14.0
Not necessary	19	11.1
Fear of hospitals	10	5.9
Forgotten	14	8.2
Not sure of the test	4	2.3
**Willingness to go for genotype test before marriage (n=171)**		
Yes	158	92.4
No	13	7.6

**Associations between respondents´ socio-demographics and knowledge of SCD:** statistically significant associations were found between age, level of education, occupation and knowledge of SCD among the respondents (p < 0.001). As the age increases, the number of respondents with good level of knowledge increases. Similarly, as the level of education increases, the number of respondents with high level of knowledge of SCD increased and higher number of respondents who were employed (69.9%) had higher level of knowledge compared to students and the unemployed (Table 5). Also, a statistically significant association existed between knowledge and awareness of self-genotype. The respondents 93(72.1%) who knew their Hb phenotype had good knowledge of SCD and premarital screening (p < 0.001) (Table 6).

**Table 5:** associations between socio-demographics and knowledge of SCD among young unmarried adults in an urban community in Lagos, Nigeria, recruited between July 2018 to January 2019 (N=300)

Demographic variables	Knowledge level		Total 300(100%)	Test of statistics
	Poor 161 (53.7%)	Good 139 (46.3%)		
**Age group (years)**				
≤19	74(67.9)	35(32.1)	109(100.0)	x2 = 16.472 p **= 0.000**
20-30	87(46.3)	101 (53.7)	188(100.0)	
≥31	0(0.0)	3(100.0)	3(100.0)	
**Sex**				x2 = 2.137 p **=** 0.144
Female	54 (48.2)	58 (51.8)	112(100.0)	
Male	107(56.9)	81(43.1)	188(100.0)	
**Level of education**				x2 = 16.895 p **= 0.000**
Primary	6(60.0)	4(40.0)	10(100.0)	
Secondary	137(59.6)	93(40.4)	230(100.0)	
Tertiary	18(30.0)	31(70.0)	60(100.0)	
**Occupation**				
Employed	25 (30.1)	58(69.9)	83(100.0)	x2 = 23.361 p **= 0.000**
Students	127(62.0)	78(38.0)	205(100.0)	
Unemployed	9(75.0)	3(25.0)	12(100.0)	
**Religion**				
Christianity	127 (53.8)	109 (46.2)	236 (100.0)	x2 = 0.010 p **=** 0.922
Islam	34 (53.1)	30 (46.9)	64 (100.0)	

**Table 6:** association of knowledge of SCD and awareness of haemoglobin phenotype among young unmarried adults in an urban community in Lagos, Nigeria, recruited between July 2018 to January 2019 (N=300)

Awareness of Hb phenotype	Knowledge level		Total 300 (100%)	Test of statistics
	Poor 161 (53.7%)	Good 139 (46.3%)		
No	125(73.1)	46(26.9)	171 (100.0)	x2= 60.395 p = 0.000
Yes	36(27.9)	93(72.1)	129 (100.0)	

## Discussion

The need to focus on the young unmarried adults as the target population for premarital screening for sickle cell disease (SCD) is very important, because their knowledge and attitude towards the disease may likely affect their choice of a life partner. The results of the present study clearly demonstrated that less than half 139 (46.3%) of the respondents had good knowledge of SCD and premarital screening. This finding is similar to a study conducted in Ile-Ife, Nigeria, which reported that more than half (69%) of the respondents had poor knowledge of SCD [7]. Another study on knowledge and attitude regarding premarital screening for SCD among students of state school of Nursing Sokoto, Nigeria found that 34.1% of respondent had good knowledge of SCD and premarital screening [13]. A study conducted in Benin City, Nigeria among senior secondary school student found only 18% with correct idea of SCD [16]. Also, a study among youth corps members in Lagos Nigeria, reported that 25.3% of respondents in the intervention and 23.5% in the control group had good knowledge of SCD and screening pre-intervention [17]. Similarly, a study among Saudi adults reported that 28.8% of the respondents had good knowledge [18]. However, this finding is contrary to a study among youths in Lagos, Nigeria which reported an average of 80% of the respondents had knowledge of SCD and premarital screening [8]. This finding calls for regular and continuous health education among the unmarried adults to improve their knowledge about SCD and premarital screening before they enter into a lifelong relationship.

This study found less than half of the respondents (38.3%) agreed that the relationship should be ended if discovered that genotype predisposes two people to having children with SCD. This finding is similar to that of a study conducted in Sultan Qaboos University, Oman, where 36% of the respondents agreed with making laws and regulation to prevent marriage between non-compatible couples [19]. A study in Nigeria, however, reported that 57.6% of the respondents felt that government should prohibit marriage between incompatible couples [13]. While another study in Nigeria concluded that a large percentage of respondents did not see SCD as important in influencing marital decisions [14]. Similarly, a study among secondary students in Nigeria reported that only 36% of the students knew the importance of premarital Hb genotype and the study encouraged premarital Hb genotype screening even at a lower level of secondary school [16]. About 67% of the respondents in this study seemed it good for everybody to know their genotype before marriage and 72% agreed that, it is important for two people in relationship to undergo genetic Counselling before marriage. However, a higher percentage (93.3%) of nursing students in Nigeria knew that premarital screening is done before marriage [13]. This higher figure compared to our study may be due to the fact that the respondents involved were nurses in training.

About half 165(55%) of the respondents in this study had positive attitude towards SCD and premarital screening. This finding is similar to a study in Nigeria where 55.4% of respondent had good attitude regarding premarital screening for SCD [13], and the study in Saudi Arabia where the best attitude was reported among 41% of the respondents [18]. However, in contrary to the study in Lagos reported where only 13.4% of the respondents showed a negative attitude towards SCD premarital Counselling [8], our study found 45% of respondents with negative attitude. This disparity in findings could be due to differences in the location of the study populations, Yaba area of Lagos where the comparism study was conducted has lots of higher institutions of learning, and it´s the home of the popular, University of Lagos, hence the respondents in that study had higher number of students with tertiary level of education 92(33%) compared to our study which was carried in highly populated area of Lagos with less number of students with tertiary level of education 60(20%). This study found that less than half of the respondents (43%) knew their haemoglobin phenotype, most (46%) of them took the test because of school entry and majority (80%) were Hb AA phenotype. The study among nursing students in Nigeria reported that 71% of respondents claimed to know their Hb phenotype and 73.1% of them claimed to be Hb AA [13]. This high figure is not a surprise among the nursing students. Studies among secondary students in Benin and Jos, Nigeria reported 32% and 59% respondents respectively who knew their haemoglobin phenotype [14,16], which are similar to our finding. A study in Ghana reported that only 34.2% of the respondents knew their sickling status and majority (50%) reported AA [20], while another study among students of three universities in Nigeria, reported that 32% did not know their own Hb phenotype [21]. The study in Ile-Ife, Nigeria found that a fourth of the married and engaged respondents did not know their partner´s sickling status [7]. This finding calls for health education about SCD and the importance of pre-marital screening among the young unmarried adults to prevent the pain, high morbidity and mortality associated with SCD. It has been suggested that SCD knowledge and pre-marital counselling be introduced early in school curriculum to prepare the minds of young adults, knowing fully well that more education is consistent with increased knowledge about SCD [20].

Some reasons such as not getting married soon (35%), didn´t know about the screening test (23%) were stated for non-screening among the respondents in this study. A study in Ghana reported that 60% of those who did not intend to find out their sickling status, was because they did not know much about the disease, while 24% said they were prepared to face anything and 16% said they did not see the need to find out [20]. The majority (92.4%) of the respondents who did not know their status in the present study, however, showed willingness to have their phenotype test done before they enter into marriage. Such positive pre-disposition towards pre-marital screening for SCD among young adults is highly encouraged for informed decision before a lifelong relationship is entered into. This finding is consistent with the study among the nursing school students in Nigeria, which reported that about 80% of respondent agreed to screen themselves and their partners before marriage [13], on the contrary, the study in Ghana reported that only 59.3% indicated positive intention to find out their phenotype status before marriage [20].

This study found a statistically significant associations between age, level of education, occupation and knowledge of SCD and premarital screening (p < 0.001). Our study found increased good knowledge of SCD and premarital screening as age, level of education increased. Similarly, most who were employed had good knowledge of SCD and premarital screening compared to the unemployed and students. This finding could be explained that, the higher the age, the closer an adult is getting into a relationship which could be long-lasting, hence the more the knowledge about premarital screening and SCD. Effect of education on knowledge of SCD has been shown in a study conducted among youth corps members in Lagos, that health education improved both knowledge and attitude of the youth corps members on SCD and screening uptake [13]. Also, the employed respondents may be more exposed to information about SCD among colleagues, hence more knowledge. A similar study among Saudi Adults found a statistically significant association between the level of knowledge and age groups of respondents (p= 0.043) [19]. The study in Lagos also reported a significant association between respondents‘ educational qualification and their knowledge of SCD and premarital screening, while sex and religion had no significant association with SCD premarital Counselling [8]. Similarly, Though, Nigeria is a very religious country, and it was anticipated that the religion might influence the respondent´s knowledge of SCD and premarital screening however, our results showed that there was no significant association between religion, sex and knowledge of SCD and premarital screening. This study also found that knowledge of respondents about SCD were statistically significant with awareness of self-haemoglobin phenotype (p < 0.001). Most of the respondents who were aware of their Hb phenotype had good knowledge of SCD and premarital screening. This is not a surprise, as most respondents who knew their Hb phenotype might have undergone health education or counselling on SCD and premarital screening before taking the test. This study was community based with strict adherence to ethical rules in data collection. The cross-sectional nature of the study does not allow for causal inferences. However, this study adds to the body of evidence of study on sickle cell disease and may be applicable to other similar community studies.

## Conclusion

This study found that less than half of the respondents had good knowledge of SCD and premarital screening, about half had positive attitude and majority had poor premarital screening practices of SCD. Community based health education programs on sickle cell disease will go a long way to improve the knowledge, attitude and premarital screening practices among the young unmarried adults. Development of policies which will ensure easy access to pre-marital counselling and SCD screening services in the community for an informed decisions and actions in the choice of partners among the young unmarried adults in order to reduce the prevalence of SCD is highly recommended.

### What is known about this topic

- SCD constitute a major public health problem among the black race;
- Nigeria has the highest prevalence of SCD worldwide with an estimate of 2.3% of the population suffering from sickle cell disorder.

### What this study adds

- The present study shows poor knowledge of and negative attitude towards SCD and premarital screening;
- The study shows that premarital screening for SCD is poor;
- The study shows the factors affecting the knowledge of SCD e.g. age, level of education and occupation of respondents.

## References

[1] American Society of Hematology State of Sickle Cell Disease Report 2016.

[2] Yusuf HR, Lloyd-puryear MA, Grant AM, Parker CS, Creary MS, Atrash HK (2011). Sickle Cell Disease. The Need for a Public Health Agenda. Am J Prev Med.

[3] Modell B, Darlison M (2008). Global epidemiology ofhaemoglobin disorders and derived service indicators. Bull World Heal Organ.

[4] Afolayan JA, Jolayemi FT (2011). Parental Attitude to Children with Sickle Cell Disease in Selected Health Facilities in Irepodun Local Government, Kwara State, Nigeria. Ethno Med.

[5] Ruiz MRM, Blanes LYB, Garcia LMC, Smith LLEF (2007). Sickle Cell Anemia and Hemoglobin SC Disease incidence rates in Havana City, Cuba from 1995 to 2004. REV CUBANA GENET COMUNIT.

[6] Bolanle O, Rotimi C, Cornelius A (2013). Stigmatizing attitude towards peers with sickle cell disease among secondary school students in Nigeria. Int J Child. Youth Fam Stud.

[7] Abioye-Kuteyi EA, Oyegbade O, Bello I, Osakwe C (2009). Sickle cell knowledge, premarital screening and marital decisions among local government workers in Ile-Ife, Nigeria. Afr J Prm Health Care Fam Med.

[8] Gabriel OO, Matthew CO (2013). Knowledge, attitude and practice of premarital Counselling for sickle cell disease among youth in Yaba, Nigeria. Afr J Reprod Health.

[9] Anie KA, Egunjobi FE, Akinyanju OO (2010). Psychosocial impact of sickle cell disorder: Perspectives from a Nigerian setting. Globalization and Health.

[10] World Health Organization (2006). Fifty-ninth World Health assemblies: Resolutions and decisions, annexes, WHA59/2006/REC/1.

[11] Adewuyi JO (2000). Knowledge of and attitudes to sickle cell disease and sickle carrier screening among new graduates of Nigerian tertiary edu-cational institutions. Niger Postgraduate Medical Journal.

[12] Affordable Reliable Web Hosting Solutions.

[13] Isah B, Musa Y, Mohammed UK, Ibrahim MTO, Awosan KJ, Yunusa EU (2016). Knowledge and Attitude Regarding Premarital Screening for Sickle Cell Disease among Students of State School of Nursing Sokoto. Annals of International Medical and Dental Research.

[14] Olakunle OS, Kenneth E, Olakekan AW, Adenike OB (2013). Knowledge and attitude of secondary school students in Jos, Nigeria on sickle cell disease. Pan African Med J.

[15] Boadu I, Addoah T (2018). Knowledge, beliefs and attitude towards sickle cell disease among university students. Journal of Community Medi-cine & Health Education.

[16] Bazuaye GN, Olayemi EE (2009). Knowledge and Attitude of Senior Secondary School Students in Benin City Nigeria to Sickle Cell Disease. World Journal of Medical Sciences.

[17] Olatona FA, Odeyemi KA, Onajole AT, Asuzu MC (2012). Effects of Health Education on Knowledge and Attitude of Youth Corps Members to Sickle Cell Disease and its Screening in Lagos State. J Community Med Health Educ.

[18] Al-qattan HM, Amlih DF, Sirajuddin FS, Alhuzaimi ID, Alageel MS, Bin Tuwaim RM, Al Qahtani FH (2019). Quantifying the Levels of Knowledge, Attitude, and Practice Associated with Sickle Cell Disease and Premarital Genetic Counselling in 350 Saudi Adults. Hindawi Advances in Hematology.

[19] Kindi RA, Rujaibi SA, Kendi MA (2012). Knowledge and Attitude of University Students towards Premarital Screening Program. Oman Med J.

[20] Foanor JA, Anthony QQ (2019). Sickle Cell Disease Awareness, Depth of Knowledge and Attitude Towards Premarital Screening Among Students in Ghana. African Journal of Management Research.

[21] Olubiyi SK, Umar JN, Ajiboye O, Olubiyi VM, Abioye TAS (2013). Knowledge and attitude of undergraduates of Ekiti State Univer-sity towards sickle cell disease and genetic coun-seling before marriage. Sky J Med Med Sci.

